# Optimal design of an electrochemical reactor for blackwater treatment

**DOI:** 10.1002/wer.1374

**Published:** 2020-07-05

**Authors:** Siva Varigala, Srinivas Krishnaswamy, Chandra P. Lohia, Meghan Hegarty‐Craver, Sonia Grego, Michael Luettgen, Clement A. Cid

**Affiliations:** ^1^ Department of Chemical Engineering BITS Pilani K K Birla Goa Campus Goa India; ^2^ ITC‐Kohler Co. Pune India; ^3^ RTI International Research Triangle Park NC USA; ^4^ Center for WaSH‐AID Duke University Durham NC USA; ^5^ Kohler Co. Kohler WI USA; ^6^ California Institute of Technology Pasadena CA USA

**Keywords:** blackwater treatment, electrode cleaning, mineral deposits, reverse polarity, uniform mixing

## Abstract

Electrolysis of blackwater for disinfection and nutrient removal is a portable and scalable technology that can lessen the need for cities to construct large‐scale wastewater treatment infrastructure and enable the safe onsite reuse of blackwater. Several systems for treating wastewater from single toilets are described in the literature, but there are few examples of systems designed to use electrolysis to treat blackwater from nearby toilets, which is a situation more common in densely packed urban living environments. In order to scale a single toilet electrolysis system to one that could service multiple toilets, computational fluid dynamic analysis was used to optimize the electrochemical reactor design, and laboratory and field‐testing were used to confirm results. Design efforts included optimization of the reactor shape and mixing to improve treatment efficiency, as well as automated cleaning and salt injection to reduce maintenance and service requirements.

**Practitioner points:**

- Design of a reverse polarity mechanism to enable in situ electrode cleaning and improve long‐term electrode performance.
- Optimization of a hopper design and drainpipe location to collect and remove flaking precipitates and mitigate maintenance issues.
- Design of an automated salt injection system to guarantee sufficient chloride levels for producing adequate chlorine residuals for consistent disinfection.

## Graphical abstract

Optimized electrochemical reactor for blackwater treatment.

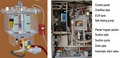

## Introduction

an estimated 4.2 billion people do not have access to properly managed sanitation systems (WHO/UNICEF JMP, 2019), which negatively affects their health and economic conditions, as well as the environment in which they live. Furthermore, 1 out of 10 persons do not even have access to a toilet and practice open defecation. This increases the likelihood of spreading diarrheal diseases and nematode infections (Prüss‐Üstün, Wolf, Corvalán, Bos, & Neira, 2016) that are responsible for high rates of childhood stunting (Spears, Ghosh, & Cumming, 2013).

In 2011, the Bill & Melinda Gates Foundation announced the launch of the Reinvent the Toilet Challenge (Kone, 2012). The intention of this Challenge was to develop technologies that would radically improve sanitation. The guidelines for the development of a Reinvented Toilet (RT) were that this RT:

– is hygienic and sustainable for the world's poorest populations,
– has an operational cost of $0.05 per user, per day,
– does not discharge pollutants, but instead generates useful energy (*e.g.,* electricity) and recovers salt, water, and nutrients,
– is designed for use in a single family home.

In response to the Challenge, Hoffmann and co‐workers developed the Caltech RT (Hoffmann et al., 2014). This RT interfaced with a traditional flush toilet. Blackwater was partially treated in a sedimentation/biological digestion tank that was followed by an electrochemical reactor (ECR), which was the core of the treatment technology. The ECR was designed as a batch reactor and sanitized waste through the generation of surface‐bound hydroxyl radicals leading to the generation of reactive chlorine species (RCS) by oxidation of Cl^−^ at semiconductor oxide anodes (Cho et al., 2014; Weres, 2009; Weres & O'Donnell, 2003). The generation of RCS leads to the removal of urea and ammonia (NH_3_ + ${\text{NH}}_{4}^{+}$) by formation of chloramines (Cho & Hoffmann, 2014) and to disinfection (Huang et al., 2016). Stainless steel cathodes balance the electrochemistry by reducing protons to H_2_. Furthermore, organic matter as chemical oxygen demand (COD) is oxidized concomitantly (Jasper, Shafaat, & Hoffmann, 2016). Thereby, the toilet effluent (urine, feces, flush water, and other human excreta) is treated, discolored, and disinfected to acceptable levels for reuse as flush water in the traditional flush toilet.

Cid *et al*. described the early design (*i.e., alpha* prototype) and field‐testing of the Caltech RT (Cid, Qu, & Hoffmann, 2018). The limitations of the *alpha* prototype testing were mostly due to the relatively low concentration of organics and nutrients in the wastewater (COD: 100 – 335 mg O_2_ L^−1^, NH_3_ + ${\text{NH}}_{4}^{+}$: 11–20 mmol/L, ${\text{PO}}_{4}^{3-}+{\text{HPO}}_{4}^{2-}$: 0.64 mmol/L) because of the relatively low usage of the *alpha* prototypes in university campus and public parks settings. Furthermore, the *alpha* prototypes were designed to treat wastewater from a relatively modest number of daily users (less than 40) with a large footprint (namely 9.3 m^2^) and a relatively limited control system.

The design of the CLASS (Closed Loop Advanced Sanitation System) presented here was based on the work of Hoffmann and co‐workers (Cho et al., 2014; Cid, Qu, et al., 2018; Hoffmann et al., 2014; Huang et al., 2016; Park, Choo, Park, Choi, & Hoffmann, 2013; Park, Vecitis, & Hoffmann, 2008) and their *alpha* prototype (vide supra). As opposed to the *alpha* prototype which was designed to treat a relatively small volume of wastewater from a single public toilet (0.2 m^3^/day), the purpose of the CLASS was to treat wastewater from several toilets in a multi‐story apartment building (*i.e*., the initial target was 5 families of 3–5 people, 0.8 m^3^/day) with a similar overall footprint.

Initially, three identical CLASS prototypes (version 1; v1) were assembled and connected to toilets in three different apartment buildings in Coimbatore, Tamil Nadu, India. Field‐testing with adequate blackwater availability was conducted for 10 months (Table S1), and the lessons learned from this effort lead to a redesign of the system (version 2; v2). Two CLASS v2 protypes were installed in the same locations as the original v1 Units A and B, and performance evaluated for 12 months.

Details on the performance testing of the two CLASS versions, overall energy estimates for the improved version, and complete description of the flow through CLASS system have been described elsewhere (Varigala et al., 2020; Welling et al., 2020).

This paper describes the design of the ECR for CLASS v1, the challenges encountered during field‐testing, and the significant modifications that were implemented in the improved CLASS v2. Major changes discussed herein include automated salt injection, the addition of a stirring mechanism, and the implementation of an automated electrode cleaning routine and optimized design to remove flaking precipitates. Computational fluid dynamic (CFD) analysis was used to inform design, and modifications iteratively tested in field and laboratory trials.

## Design

### System installation description

CLASS prototypes were installed at the ground level outside of two separate apartment buildings in Coimbatore, Tamil Nadu, India. A detailed description of the flow through the CLASS system has been published elsewhere (Varigala et al., 2020). In each building, the toilet effluent (blackwater) was piped separately from the rest of the apartment's wastewater pipes. The blackwater from 12 toilets was collected in a sump and pumped to settling tanks in the CLASS. Biological pretreatment partially clarified the blackwater and reduced the nutrient and organic load. Disinfection was achieved using electrochemical processes, and the water from the ECR was pumped through a microfiltration system to remove remaining suspended solids. The treated water was stored in a treated water tank on the rooftop of the apartment building and recycled as flush water when the system was operating in “closed loop.”

### Design of ECR in CLASS v1

The *alpha* prototype was used as the basis of design for the CLASS v1 ECR. The *alpha* prototype was designed to handle an average of 15–20 toilet flushes per day (0.2 m^3^), and treated blackwater in batches (*V*_ECR_ = 22 L). Additionally, the system operated on the campus of the California Institute of Technology (Caltech) in Pasadena, California USA, where electricity was uninterrupted. In contrast, the CLASS unit was designed to handle at least 100 toilet flushes per day (0.8 m^3^) and treat blackwater in batches as available, in an environment where power outages are common (*i.e*., a daily power availability *P_A_* = 0.8 was assumed (Min, O'Keeffe, & Zhang, 2017)). The reactor volume and electrode surface area were defined according to these requirements (*i.e*., volume to surface ratio, *r_vs_*) and assuming a constant current for a fixed treatment time:

(1) $$\left(\begin{array}{c} \left(a\right)\;TC_{\mathrm{ECR}}=\frac{r_{\mathrm{VS}}}{\tau_{\mathrm{ECR}}}=\frac{V_{\mathrm{ECR}}}{S_{A}\times \tau_{\mathrm{ECR}}}\\ \left(b\right)\;TC=TC_{\mathrm{ECR}}\times P_{A} \end{array}\right\}r_{\mathrm{VS}}=\frac{TC\times \tau_{\mathrm{ECR}}}{P_{A}}$$

where *TC_ECR_* is the treatment capacity of the ECR, *V_ECR_* is the volume of wastewater that needs to be treated, *S_A_* is the surface area of the electrodes, and *τ_ECR_* is the treatment time.

The *alpha* prototype ECR featured a relatively large water volume (*V_ECR_* = 22 L) to electrode surface area (*S_A_* = 1.8 m^2^) ratio *r_VS_* = 12 L/m^2^, requiring the use of a circulation pump to run continuously during treatment to avoid dead zones. A 4‐hr treatment time was needed to achieve complete disinfection and satisfactory COD and NH_3_ + ${\text{NH}}_{4}^{+}$ removal (Cid, Qu, et al., 2018). This resulted in a treatment capacity TC = 3 L m^−2^ h^−1^ with power available all day (*P_A_* = 1).

The CLASS v1 was expected to treat wastewater with similar properties to the *alpha* prototype using electrodes with the same formulation (*i.e*., TC was assumed to be the same). In order to accommodate the larger volume of wastewater to treat and the lower power availability that was anticipated (*P_A_* = 0.8), the target treatment time was reduced from 4 hr to 1.5 hr. Using Equation (1), *r_VS_* was calculated to be at least 5.625 L/m^2^. This target was met by selecting *V_ECR_* = 55 L and *S_A_* = 9.4 m^2^ (actual *r_VS_* = 5.8 L/m^2^), and this configuration was achieved by tightly placing 7 electrode sets in the treatment tank. There was minimal space between the bottom of the electrode arrays and the bottom of the tank, so no stirrer was used.

### Design of ECR in CLASS v2

An improved biological pretreatment subsystem was added to the CLASS v2 prototypes to increase COD and the NH_3_ removal prior to electrolysis (Varigala et al., 2020), and the ECR was redesigned based on this reduced treatment load. The number of electrode stacks was reduced from 7 stacks (*S_A_* = 9.4 m^2^) to 2 stacks (*S_A_* = 2.5 m^2^), and the effective treatment volume was increased to *V_ECR_* = 62 L. This resulted in *r_VS_* = 25 L/m^2^ for the CLASS v2 prototypes compared with *r_VS_* = 5.8 L/m^2^ for CLASS v1. With a much larger volume of water surrounding the electrodes, a stirring function was added to the ECR and the dimensions of the electrodes were modified to enable uniform mixing in the ECR tank during treatment. The electrochemical treatment was run continuously in 2‐hr batch processes (70 L of ECR total tank volume) depending upon the availability of wastewater. A total of 842 ± 171 L of wastewater were treated daily at Site A, and 1,074 ± 257 L of wastewater were treated daily at Site B (Varigala et al., 2020).

### Improvements implemented in CLASS v2

Several design changes were made between the v1 and v2 CLASS prototypes to address the operational challenges experienced during the initial phase of field‐testing including:

- integration of an automated salt injection system to maintain adequate chloride concentration,
- addition of a mechanical stirring mechanism to ensure uniform mixing of reactor contents,
- incorporation of an in situ method for electrode cleaning by reverse polarization to address the heavy mineral scale,
- redesign of the ECR tank to include a hopper (vs. flat bottom) to collect precipitates so that they could be periodically pumped out.

These changes were iteratively tested through a combination of computer‐aided design (CAD), simulations, and field trials.

## Methodology

### Salt addition

**Figure 1:**
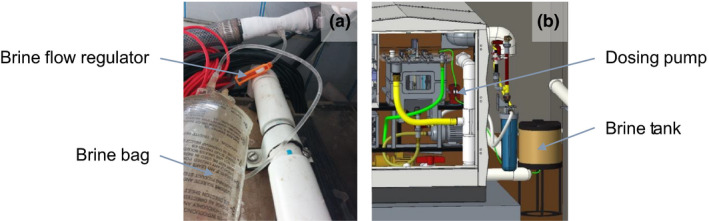
(a) Custom venturi prototype with reusable saline bag and flow regulator. (b) Automated salt injection system CAD with dosing pump and saturated brine tank.

Sodium chloride (NaCl) in the form of common table salt was added to the blackwater to guarantee a minimum Cl^−^ concentration that enabled consistent disinfection with adequate chlorine residuals (free chlorine >1 mg Cl_2_/L) in the treated water tank. Three methods of salt addition were tested: (a) manual batch pouring of salt prior to the blackwater entering the ECR tank, (b) passive addition using a custom venturi prototype with reusable saturated saline bag and flow regulator (Figure 1a), and (c) automated brine injection using a solenoid‐operated diaphragm pump (ANT, ED‐01) with adjustable speed (Figure 1b). For methods 2 and 3, the saturated salt solution was added to the ECR before the start of a treatment cycle.

### Automatic electrode cleaning

An in situ periodic electrode cleaning regimen was developed for the CLASS v2 prototypes to reduce the maintenance requirements. A dedicated power supply (HRP‐450–7.5 V, Meanwell) was used for the reverse polarization scheme whereby half of the stainless steel cathodes were energized with a positive potential and the other half with negative potential for the first half of the cleaning cycle (approx. 10 min), and the respective polarities reversed for the second half of the cleaning cycle. No significant oxidation of stainless steel cathodes was observed during the cleaning cycle. The stainless steel cathodes were not replaced during the field‐testing period. The frequency of cleaning cycles was kept to a minimum (*i.e*., 1 cleaning cycle per 50 electrolysis cycles) to minimize risk of corrosion of the stainless steel cathodes.

Further, no voltage was applied to the semiconductor oxide electrode (anodes) during the cleaning mode, thereby the cleaning procedure did not affect the RCS species generation ability of the electrochemical system. Also, no additional maintenance need was observed for the semiconductor oxide anodes after the reverse polarization scheme was implemented. The precipitates flaking off the stainless steel cathodes were automatically evacuated through an electric drain valve (MDB‐2, Depend‐o‐Drain). The cleaning function was integrated in the logic operating the system and automatically implemented with a fresh batch of blackwater after a fixed number (typically 50) of electrolysis cycles.

### Collection of flaking mineral scale

**Figure 2:**
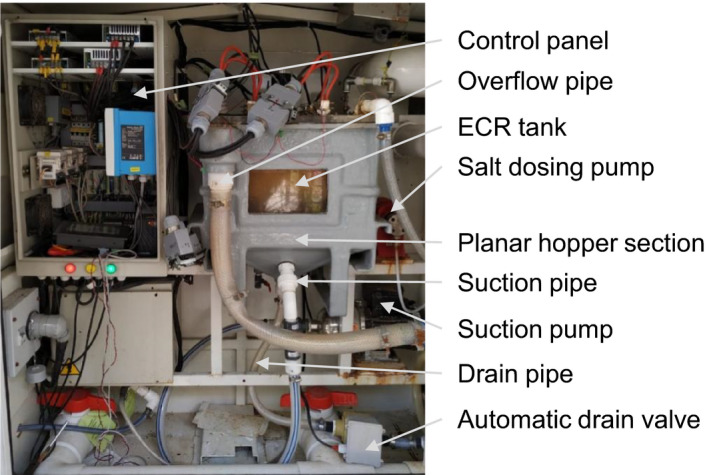
Arrangement of the suction pipe to remove the treated water, the drainpipe to expel the settled precipitates, and the auto drain valve.

The CLASS v1 ECR had a flat bottom tank which resulted in the accumulation of flaking mineral scale from the stainless steel electrodes surface over time (Figure S1). Additionally, some of these precipitates were resuspended in the electrochemically treated water contributing to its cloudy appearance (Figure S2). The CLASS v2 ECR was redesigned with a planar hopper section at the bottom of the tank that served to collect the flaking mineral scale (Figure 2). The precipitates were periodically flushed from the hopper through a drainpipe. The planar hopper angle with the vertical plane of the tank was designed based on the inputs obtained from the standard design charts of Schulze (Schulze, 2008) (Figure S3). The drainpipe was sized to ensure that the precipitates would flow freely through the drain opening.

### Simulation methods

#### Suction pipe design at the hopper bottom

This simulation study was envisaged to design an optimum mechanism to frequently collect and remove the precipitates from the ECR tank in order to increase the meantime between maintenance tasks. Simulations were also performed in Ansys 17.1 (Ansys) to determine the optimal location of the suction pipe in the hopper bottom to minimize the carry‐over of the settled solids when treated water was removed from the tank while maintaining a minimal buffer layer of fresh water above the settled solids.

**Figure 3:**
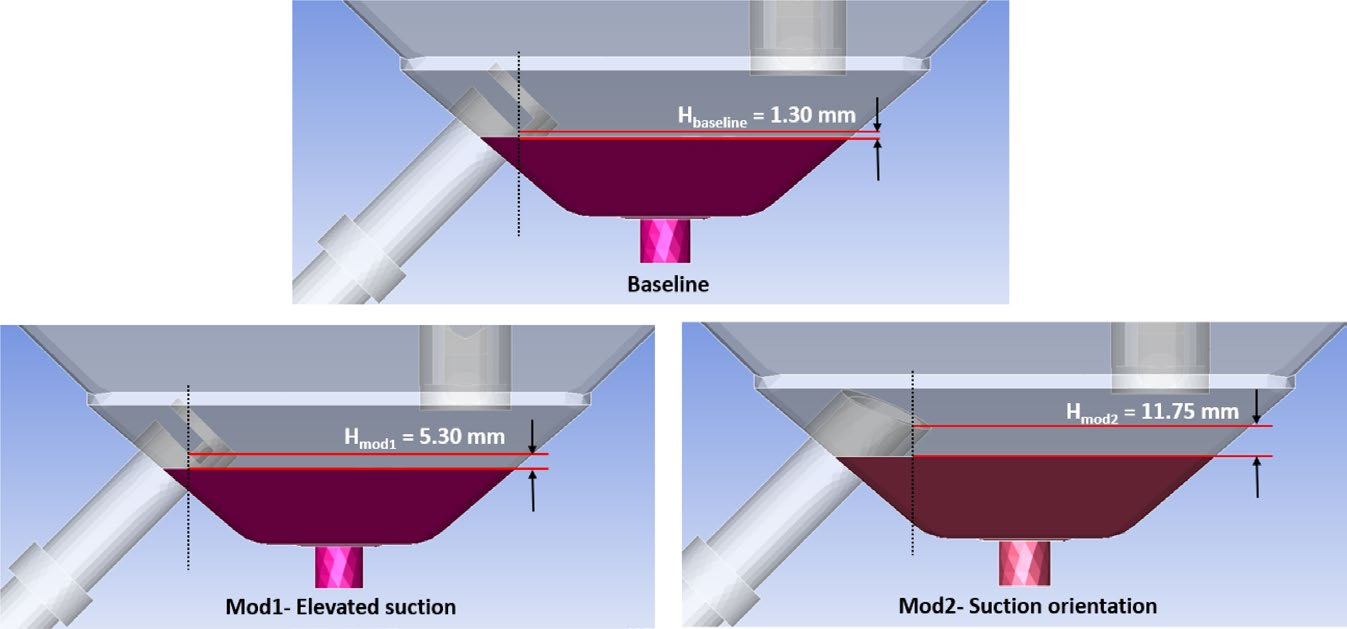
CFD geometries of the simulated models for hopper design. Suction pipe (white) and drain pipe (purple) are represented.

Based on the theoretical estimates, a 3D planar hopper model (Figure 3) with two inlets open to atmosphere and one outlet with suction pressure was developed to optimize the location and design of suction pipe such that solids and sediments do not carry‐over into the treated water.

The height of the sediment zone in the hopper should ideally be as low as possible (since it adds to the dead volume in the ECR tank) while allowing enough volume to cater for the settled precipitates. For the simulation studies, initially a height of 30 mm from bottom was considered as sediment zone and the rest of the volume was patched as water. Further, varying inlet height, H from the top of sediment zone as shown in Figure 3 was simulated to minimize the buffer layer volume that also adds to the dead volume of the reactor (sediment zone).

The selected domain was discretized into several small cells for calculations. A transient modeling approach was adopted since the sediments (solids slurry) and water level in the tank varied over time. Gravity was applied in the vertically downwards direction.

The reactor contains both solids slurry and water which makes it appropriate to use a volume of fluid (VOF) model. VOF is applicable to a fixed Eulerian mesh and can track the interface between two or more immiscible fluids. It has surface tension and wall adhesion force modeling capability for solving different phase volume fraction equations. A single set of momentum equations is shared by the fluids. The volume fraction in each discretized cell adds up to unity. From the volume fraction, a volume‐averaged value is calculated for each field variable and property at each iteration. To track the interface between the two phases in the hopper section, the continuity equation is solved for each phase. For water (denoted as subscript *w*), the equation is as follows (Equation 2):

(2) $$\frac{1}{\rho_{w}}\left[\frac{\partial }{\partial t}\left(\alpha_{w}\rho_{w}\right)+\nabla \cdot \left(\alpha_{w}\rho_{w}{\vec{v}}_{w}\right)\right]=S_{\propto_{w}}+\left({\dot{m}}_{\mathrm{aw}}-{\dot{m}}_{\mathrm{wa}}\right)$$

where, *a*: solids slurry; *w*: water; *ρ*: density; *α*: volume fraction; $\vec{v}$: flow velocity; $S_{\propto_{w}}$: mass source for water phase, equal to zero for this case; ${\dot{m}}_{\mathrm{aw}}$: mass transfer from slurry to water; ${\dot{m}}_{\mathrm{wa}}$: mass transfer from water to slurry.

Water volume fraction is computed using the following equation (Equation 3):

(3) $$\alpha_{w}+\alpha_{a}=1$$

As for the momentum, only one equation (Equation 4) will be solved for the entire domain and it will be dependent on the volume fractions:

(4) $$\frac{\partial }{\partial t}\left(\rho \vec{v}\right)+\nabla \cdot \left(\rho \vec{v}\vec{v}\right)=-\nabla p+\nabla \cdot \left[\mu \left(\nabla \vec{v}+\nabla {\vec{v}}^{T}\right)\right]+\rho \vec{g}+\vec{F}$$

where ∇*p*: gradient of pressure; *µ*: dynamic viscosity; $\vec{g}$: acceleration due to gravity; $\vec{F}$: external forces acting on the system, equal to zero for this case.

The differential equations were discretized and solved iteratively based on the Newton Raphson method. Appropriate convergence criteria were applied for the continuity and momentum equations. The specific details for simulation are highlighted in Table 1.

**Table 1:** Simulation parameters

Mesh count	Number of tetrahedral elements: 5,427,875
Models	k‐ε Realizable method with standard wall function Multiphase Volume of Fluid (VOF) module
Solver	PISO scheme is used for pressure–velocity coupling
Convergence	10^–3^ tolerance for continuity and momentum

Simulations were performed in Ansys 17.1 with appropriate models and boundary conditions (vide supra). Once satisfactory convergence was reached as specified in Table 1, the results were obtained.

#### Stirrer location for uniform mixing

A stirrer was added to the ECR tank for uniform mixing of salt contents to avoid dead or stagnant zones (Palmas et al., 2018; Torotwa & Ji, 2018). Prior to the start of the CFD study, the search for simple design and readily available off the shelf stirrer blades informed the selection of two different stirrer types for comparison.

Also, the stirrer design included requirements of operation at relatively low speeds, suitable for sewage type liquids and low energy consumption. Models of different types of stirrers of similar dimensions (with length to width ratio of 1) were prepared and simulations were run, with stirrer placed at two different locations inside the tank to study the mixing distribution.

While ensuring the reactor contents were uniformly mixed, it was essential to keep the stirrer speed low to minimize the wearing of the moving parts. For this study, all the simulation studies for the two different stirrers were carried for a constant rotational speed of 200 rpm.

In Ansys Fluent 17.1, the Moving Reference Frame (MRF) approach was used to model the rotational stirring motion of the stirrer (Torotwa & Ji, 2018). This modeling approach is a *steady‐state* approximation of the comprehensive model at an *instance* of time, as the comprehensive model is highly computationally intensive. The body/mesh is not physically rotated. MRF is equivalent to running a rotational simulation and then observing the results at the instant equivalent to the position of the rotor within the MRF. MRF rotation is defined by rotation center, rotation axis, and angular velocity.

Since the interaction between the water and stirrer was relatively simple, the transient effects are not high and hence MRF model can be used. In this model, individual cell zones can be assigned different rotational and/or translational speeds. The equations were solved in their stationary forms if the zones were steady, which would happen for dead/stagnant zones. The Navier–Stokes equations for moving reference frame were obtained by transforming the stationary frame Navier–Stokes equations to a rotating reference frame. The relative velocity was used as the dependent variable in the momentum equations, and the relative total internal energy was used as the dependent variable in the energy equation.

For the relative velocity formulation, the governing equations of fluid flow for steadily rotating frame can be written as:

- Conservation of mass:

(5) $$\frac{\partial \rho }{\partial t}+\nabla \cdot \rho {\vec{v}}_{r}=0$$

- Conservation of momentum:

(6) $$\frac{\partial }{\partial t}\left(\rho {\vec{v}}_{r}\right)+\nabla \cdot \left(\rho {\vec{v}}_{r}{\vec{v}}_{r}\right)+\rho \left(2\vec{\omega }\times {\vec{v}}_{r}+\vec{\omega }\times \vec{\omega }\times \vec{r}\right)=-\nabla p+\nabla \cdot {\overset{=}{\tau }}_{r}+\vec{F}$$

- Conservation of energy:

(7) $$\frac{\partial }{\partial t}\left(\rho E_{r}\right)+\nabla \cdot \left(\rho {\vec{v}}_{r}H_{r}\right)=\nabla \cdot \left(k\nabla T+{\overset{=}{\tau }}_{r}+{\vec{v}}_{r}\right)+S_{h}$$

The momentum equation has two additional terms.

Coriolis acceleration

(8) $$\left(2{\vec{\omega }}_{r}\times {\vec{v}}_{r}\right)$$

Centripetal acceleration

(9) $$\left(\vec{\omega }\times \vec{\omega }\times \vec{r}\right)$$

The energy equation is written in terms of relative internal energy *E_r_* and relative total enthalpy *H_r_*.

- Relative internal energy:

(10) $$E_{r}=h-\frac{p}{\rho }+\frac{1}{2}\left(\upsilon_{r}^{2}-\upsilon_{r}^{2}\right)$$

- Relative total enthalpy:

(11) $$H_{r}=E_{r}+\frac{p}{\rho }$$

These equations were solved iteratively, and Figure S4 shows one of the stirrer geometries simulated.

The turbulence intensity in the water volume can be compared for mixing characteristics between the two stirrer designs. Turbulence intensity is a measure of the turbulence level and circulation intensity within the system. For a fully developed pipe flow, the turbulence intensity is given by:

(12) $$I=0.16{\left(Re_{\mathrm{dh}}\right)}^{-1/8}$$

where *Re_dh_* is the Reynolds number calculated for the pipe hydraulic diameter (*d_h_*). For a fluid that stands still (like the one here) with a stirrer for circulation, typical turbulence intensity values are below 1%. The higher the turbulence intensity, better will be the mixing.

#### Experimental validation of stirrer simulation

The CFD simulation results were experimentally validated using a transparent laboratory test rig with two acrylic blocks that were similar in size to the electrode stacks. The stirrer was connected to a motor to rotate the stirrer blade and generate the mixing effect. The tank was filled with tap water and fluid movement trackers (*i.e.,* pieces of colored paper) to visually observe the dead zones and the mixing distribution inside the tank for a given rotor speed. Further, methylene blue dye was used to observe the mixing gradients of the color in the water due to stirrer rotation.

### Analytical methods

Electrical conductivity (EC) was measured using a handheld conductivity meter (Myron L) and used as a loose proxy for measuring the effect of Cl^−^ addition. Cl^−^ content was periodically measured by a certified third‐party laboratory according to method IS 3025 (Part 32) 1998.

The composition of the deposited mineral content on the electrode surface was analyzed using X‐ray powder diffraction spectra (Philips PANalytical X'Pert Pro X‐ray) technique.

Calcium (Ca^2+^) content in wastewater was measured by a third‐party water analysis laboratory according to method IS 3025 (Part 40) 1991 for sites A, B, and C, and ion chromatography (Dionex ICS 2000; AS19G anions, CS12A cations) for the sample from Caltech.

## Results and discussion

### Table salt (NaCl) addition

**Figure 4:**
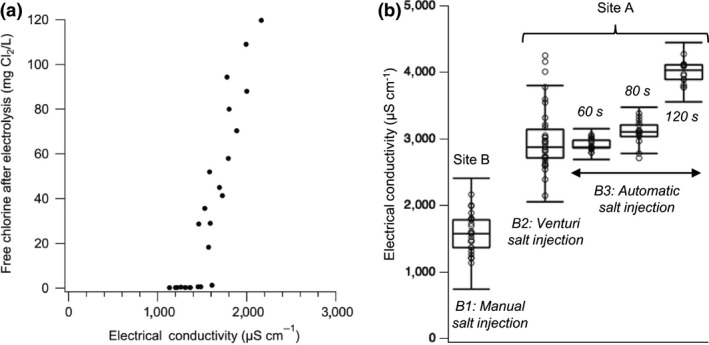
(a) Observed relationship between free chlorine concentration in the treated water and electrical conductivity (EC) after electrolysis—data from site B. (On average, EC decreased between 500 and 600 µS/cm during electrolysis due to aqueous chlorine formation.) (b) Treated water EC for three different salt injection methods (B1: Manual, B2: Venturi, and B3: Automatic, for 60 s, 80 s, and 120 s dosing time). Each data point represents a daily measurement. The top and bottom hinges of the box plot represent the highest (Q3) and lowest (Q1) quartile, respectively. The middle segment represents the median treated EC value. The bottom inner fence represents Q1 − 1.5 * (Q3 − Q1) and the top inner fence represents Q3 + 1.5 * (Q3 − Q1).

The blackwater EC received from the CLASS system was relatively unchanged over the testing period with daily variations of ±300 µS/cm, although properties differed between sites depending on the mineral content of the pour flush and the personal wash water sources used. At site A, the pour flush and the personal wash water source was fed by borewell water, resulting in the EC of the blackwater of 3,162 ± 221 µS/cm (*n* = 36) and a Cl^−^ content of 424 ± 80 mg/L (*n* = 9), where “*n*” stands for the number of samples analyzed. At site B where the pour flush and the personal wash water source was fed by RO water, the EC of the blackwater was 1,600 ± 290 µS/cm (*n* = 11) and the Cl^−^ content was 188 ± 68 mg/L (*n* = 11). It has been shown that sufficient Cl^−^ concentration (>350 mg/L) in the blackwater is essential to electrochemically generate sufficient aqueous total chlorine (>5 mg Cl_2_/L) for disinfection of pathogens with COD and NH_3_ removal (Cho & Hoffmann, 2014; Huang et al., 2016). The same was observed in this study: free and total chlorine concentrations after electrolysis increased rapidly as a function of EC after a threshold of 1,600 µS/cm in treated water was reached on site B (Figure 4a). The solution conductivity was reduced by 500–660 µS/cm after electrolysis due to the oxidation of Cl^−^ into non‐ionic RCS. Therefore, to ensure consistent chlorination, Cl^−^ concentrations at site A and especially at site B was increased by NaCl addition in the form of the table salt.

Different approaches were evaluated to add salt to blackwater prior to treatment. Initially, when the CLASS was in “opened loop,” salt was added manually to the cistern containing freshwater for flushing the toilets. The lag time between salt addition and an associated measurable response in EC at the CLASS settling tanks was on the order of 1–2 days, which made it difficult to adjust chloride to the desirable level. Salt addition was then shifted to direct addition to the settling tanks via manual addition (site B and C, Figure 4B1) or a venturi mechanism (site A, Figure 4B2).

In the manual addition set up of site B where RO water was used, 4.6 kg NaCl/week (*n* = 46) was added to the settling tank (approximately 650 g/1,000 L). Figure 4B1 shows the fluctuations in EC associated with manual addition of salt to the blackwater in the settling tanks at site B. Closing the loop by connecting treated water to cistern flushing did not eliminate the need for regular salt addition because of the extensive use of fresh water from the personal cleaning tap and continued practice of pour‐flushing as studied by Welling and co‐workers (Welling et al., 2020).

In the venturi mechanism configuration tested at site A, a venturi tube connected to a bag of saturated saline solution was added in line to the pipe feeding the ECR tank so that salt was added every time the reactor filled with wastewater. This configuration had the advantage of providing passive salt dosing with a reservoir lasting through 2 + days of operation. Unfortunately, the system clogged frequently (typically at least once per week) and dosing was not uniform (Figure 4B2).

Based on lessons learned from the venturi configuration, an active brine injection mechanism was designed for the CLASS v2 prototypes. This pump‐based active dosing system provided highly automated uniform brine injection, and EC values remained steady to within ±185 µS/cm for up to a month (Figure 4B3). Furthermore, controlling the dosing time of the injection pump enabled predictable changes of EC for three different dosing times of 60 s, 80 s, and 140 s.

### Uniform mixing of solution in ECR tank

#### Bubble‐driven agitation in CLASS v1

**Figure 5:**
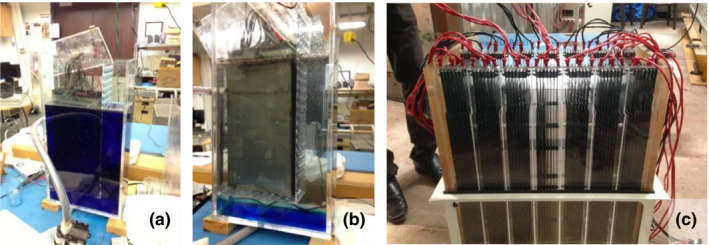
Feasibility determination of tightly packing the electrode sets in the ECR tank. (a) Single electrode set in tank (*r_VS_* = 12 L/m^2^, *V_ECR_* = 22 L) filled with water colored with methylene blue dye. (b) Same setup after several minutes of electrolysis at 3.5 V. (c) Final design for the ECR tank with close packed electrode sets (*r_VS_* = 5.8 L/m^2^, *V_ECR_* = 55 L).

Water currents due to gas bubbles forming and detaching from the electrode surfaces were expected to be adequate to mix the water around and between the electrodes and minimize chlorination gradients in the ECR. Figure 5a,b shows the effective zone of treatment of an electrode pack in preliminary tests. A single electrode set in tank (*r_VS_* = 12 L/m^2^, *V_ECR_* = 22 L) was filled with water, and methylene blue dye was added as electrolysis indicator. After several minutes of electrolysis operating close to the actual field‐testing parameter of 3.5 V, the dye was oxidized, and the water had cleared in the region around the electrodes, but blue dye still remained under the electrodes where bubbles were absent. This test indicated the feasibility of an ECR without recirculation of the water under treatment if the electrodes were closely packed to the sides and bottom of the ECR tank. Thereby CLASS v1 was designed with 7 electrode sets tightly packed in a treatment tank with an electrode spacing of 3 mm and with as little space as possible at the bottom of the electrode arrays (Figure 5c).

#### Stirring in CLASS v2

While the volume of ECR tank remained approximately equal between the CLASS v1 and v2 prototypes, the number of electrode arrays was reduced from seven to two. This created the possibility of “dead zones” in the ECR tank during the treatment process requiring mechanical mixing of the reactor contents during treatment. CFD simulations informed the design and placement of a stirrer mechanism.

**Figure 6:**
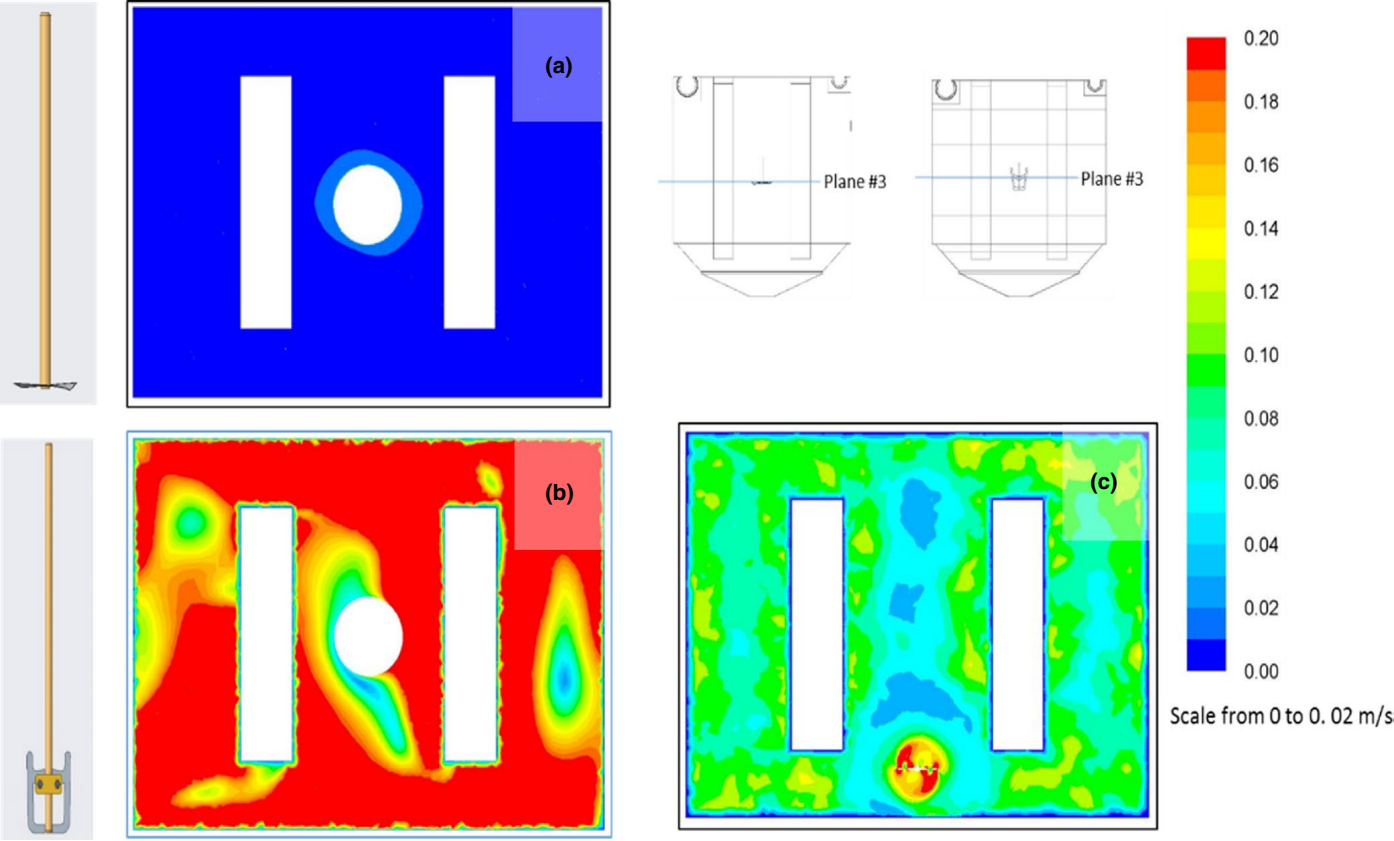
Velocity profiles for the two stirrer models: T‐shaped (a) and U‐shaped (b and c) at stirrer speed of 200 rpm located in the center of the ECR (b) and toward the front (c).

The simulation results predicted that the U‐shaped stirrer had a better mixing distribution than the T‐shaped stirrer for a given stirrer speed of 200 rpm (Figure 6a,b). The U‐shaped stirrer had low velocities of around 0.02–0.2 m/s observed between the electrode stacks at some locations with a volume average velocity of approximately 0.248 m/s while the T‐shaped stirrer had very low mixing velocities of around 0.02–0.04 m/s with average volume velocity of 0.004 m/s, indicating the presence of significant stagnation zones at several planes. Additionally, the U‐shaped stirrer provided a volume average turbulent intensity ~30% higher than the T‐shaped stirrer.

The velocity profiles indicated that the U‐shaped stirrer produced a uniform mixing result around the impeller regions as seen by the evenly distributed patterns. The extended surface area on the stirrer blade was associated with increased contact area and inducing circular flow in the fluid. The average velocity range suggests that there was relatively significant turbulence generated and thus expected to distribute the fluid more throughout the entire volume.

#### Stirrer location and experimental validation

Further for the U‐shaped stirrer, a simulation study was conducted to compare the stirred center location to a different location which is adjacent to the reactor wall, for the same stirrer speed of 200 rpm (Figure 6b,c). The mixing distribution for the center location proved more uniform spreading in all planes as compared to wall adjacent location and no additional stirrer location was evaluated.

**Figure 7:**
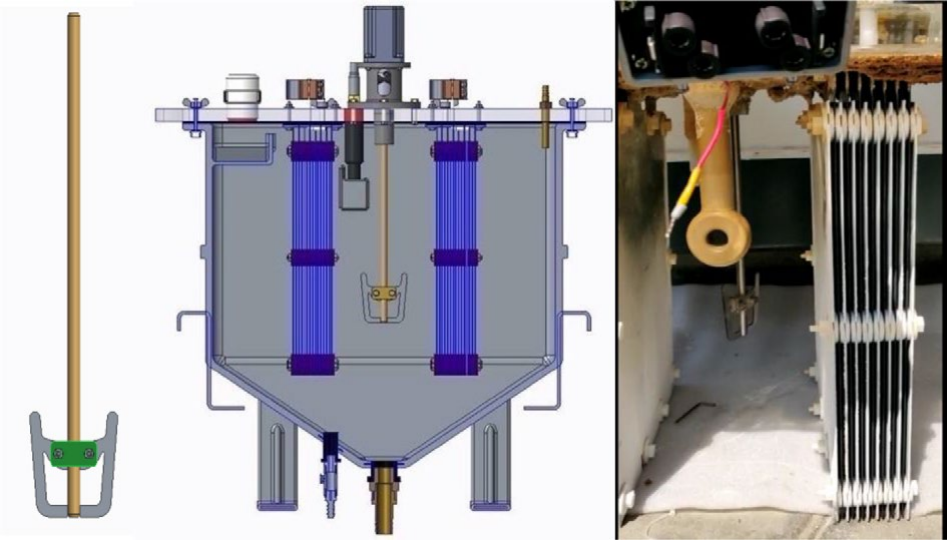
Pictorial representation of stirrer and assembly with the ECR tank top and the actual stirrer image to the right.

The velocity profiles for the U‐shaped stirrer at center location drove the fluid toward the electrode array, which then splashed back and was directed vertically and in opposite directions toward the center of the tank. The simulation was validated in laboratory scale tests. A stainless steel anchor type paddle with a length to width ratio of 1.08 was mounted on a stainless steel stirrer rod (length: 360 mm, diameter: 10 mm; Figure 7) and the stirrer rod was mounted to a high torque hybrid stepper motor (BH60 SH 65‐2804 AF – IP 65) with rotation speed of 200–300 rpm. The tracers indicated no dead zones and uniform mixing inside the reactor for the U‐shaped electrode located in the center of the tanks in agreement with the CFD results (Figure S6).

### Mineral scaling

#### High mineral scaling in CLASS v1

Mineral scaling of the electrodes due to operation of the ECR was observed during the CLASS v1 field‐testing in all three test sites but with different degrees of severity. The high calcium content of water sources particularly at sites A and C led to blackwater containing high levels of calcium (Table 2). Thus, a thick layer of white solids identified as mostly calcium carbonate (Figure S1) deposited on the electrodes during the operation of the ECR as well as flaked out filling the reactor with debris.

**Table 2:** Calcium content (mg/L) of incoming water correlates with the observed amount of sedimentation in the ECR

Site	Cistern flush water	Pour flush water	Blackwater	Treated water	Sedimentation
	Calcium concentration (mg/L)				Observation
A	9.2	169.5	89	44	Medium
B	1.2	1.2	13	5	Low
C	9.1	169.5	140	55	Heavy
^a^Caltech	‐	‐	25–44	‐	Low

^a^ Alpha prototype installed at the California Institute of Technology, Pasadena, CA 91125 USA.

Scaling or cathode fouling (*i.e.,* deposition of minerals on the surface of the electrodes) is a well‐known issue for electrochemical water treatment (Cid, Jasper, & Hoffmann, 2018; Wendt & Kreysa, 1999). This phenomenon was readily observed within days of deployment of the CLASS v1 prototypes in India. This scaling resulted in extensive (catastrophic) maintenance demands on two fronts: manual cleaning of the electrodes and ECR, and filter maintenance during which the CLASS system was inoperative. During manual cleaning, the electrodes and ECR tank were washed with tap water to remove mineral deposits—a cumbersome and time‐consuming procedure. During the filter maintenance, the polishing filters and plumbing elbows that frequently clogged were rinsed of debris, and the filter was changed if necessary. Both manual cleaning and filter maintenance interrupted operation for 1 day or more and occurred when system performance was degraded, we defined these as catastrophic maintenance events.

Maintenance requirements for the v1 prototypes were found to be related to the mineral content of the pour flush and personal wash water (especially dissolved calcium, Table 2). After electrolysis, the calcium levels in the treated water were significantly reduced in agreement with the observed levels of mineral scaling (Table 2). At site B, where the calcium levels were the lowest, mean time between maintenance events was the longest with reactor cleanings conducted approximately monthly, and the polishing filter was changed every 49 days on average (Table 3). At site A where calcium levels were higher, more frequent maintenance was needed (*i.e*., 10 days/reactor cleaning and 8 days/filter change). Scaling was most severe at site C where the blackwater calcium levels were highest. For example, over a 6‐day run period (4,690 L of treated water), 573 g of dried mineral scaling (mostly as calcium carbonate, Figure S5) that flaked off the cathodes was collected (Figure S1). The flaking also resulted in a cloudy appearance of the treated water after electrolysis (Figure S2). The longest continuous run time of the unit was less than a week because the filters clogged and the plumbing was frequently clogged, sometimes daily, with debris.

**Table 3:** Mean time between maintenance tasks for sites A, B, and C for different configurations of the systems

Time between maintenance tasks (days)	Site	A	B	C
Version	Maintenance task			
V1	Filter maintenance	10^a^ (0)^b^	49^a^ (0)^b^	5^a^ (0)^b^
	Reactor cleaning	8	29	23
V1 with recirculation	Filter maintenance	15^a^ (0)^b^	–	4^a^ (0)^b^
	Reactor cleaning	13	–	17
V2 with polarity reversal and hopper‐suction pipe	Filter maintenance	0^a^ (33)^b^	0^a^ (33)^b^	–
	Reactor cleaning	105	120	–

^a^ Catastrophic maintenance.

^b^ Preventative maintenance.

#### Solutions to manage electrode scaling

Multiple approaches were evaluated to address the scaling issue. First, a recirculation system was added to the v1 prototypes that were operating at sites A and C. The recirculation system included an additional pump and inline filters added to the reactor tank to control the mineral precipitate. This recirculation system increased the interval between maintenance events at site A (*i.e*., 15 days for filter maintenance and 13 days between reactor cleanings; Table 3); however, more frequent maintenance was needed at site C (due to relatively high mineral content) to prevent clogging of the multiple inline filters (*i.e*., every 4 days; Table 3).

Then, a radically different approach to control electrode scaling was implemented in the CLASS v2 prototypes. An in situ electrode cleaning scheme using reverse polarity was implemented (Jin, Yu, Zhang, Yan, & Chen, 2019; Lee, Hong, & Moon, 2012). Additionally, the ECR tank was designed with a hopper (versus. flat) bottom to funnel flaking mineral scale, and a suction pipe mechanism to remove treated water without the carry‐over of settled precipitates was designed with the aid of Ansys simulations.

**Figure 8:**
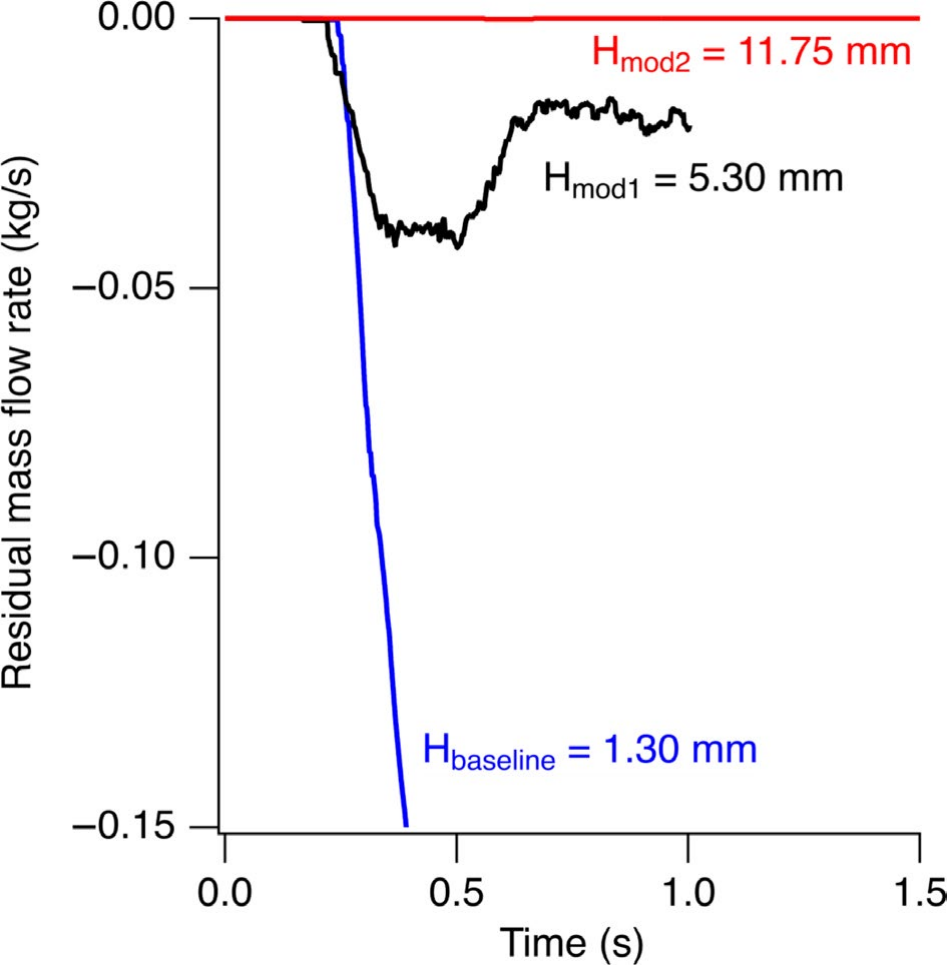
250‐point average of the calculated residual mass flow rate of sediments as a function of time for the three geometries described in Figure 3.

Figure 8 shows the plot of sediment mass flow rate as a function of time. A negative value of the flow rate indicates that the flow of sediments is being sucked out into a filter when the ECR tank is emptying, whereas the desirable outcome is to minimize the flow rate. Simulation results highlighted a high amount of sediment flow to the outlet for the baseline model described in Figure 3. This sediment flow rate kept increasing with time.

Mod1 with elevated suction (Figure 3) was simulated and resulted in an improvement over the baseline design, reducing the sediment carry‐over (Figure 8). In Mod1 design, part of the suction pipe was blocked, equivalent to a vertical height of 5.3 mm over the sediment surface. The simulation showed a comparatively reduced sediment overflow to the outlet with some flow of sediments being sucked out.

Mod2 with an improved suction orientation (Figure 3) showed no sediment flow to the outlet (Figure 8). Analytical calculations suggested that approximately 0.5 L of fresh water remained in the ECR after drainage, corresponding to the volume between the sediments and the suction pipe inlet level.

Therefore, it can be inferred that the optimum level for the suction pipe such that there is no sediment overflow was somewhere between the heights simulated in Mod1 and Mod2. However, the fact that the volume of untreated water in Mod2 (equal to ~ 0.5 L) was only 0.65% of the water volume in the ECR tank did not warrant further optimization simulations. As a result, Mod2 geometry was incorporated into the CLASS v2 ECR.

The implementation of reverse polarity cleaning along with the ECR tank shape modifications including the hopper and suction pipe mechanism significantly decreased the maintenance requirements of the CLASS v2 prototypes (Table 3). The time intensive manual reactor cleaning and forced “catastrophic maintenance” (C) procedure was never conducted during the entire 12‐month period of v2 field‐testing; however, electrodes sets were exchanged for testing purposes, so a rinsing of the reactor was carried out resulting in mean time between reactor cleanings of 105 days for site A and 120 days for site B. Polishing filters were changed at both sites A and B after 400 treatment cycles (approximately monthly) as “preventative maintenance” (P).

## Conclusion

The optimal design of an ECR for blackwater treatment described in this paper is the result of modeling efforts and design improvements that overcame the significant operational challenges that were encountered during field‐testing. The optimal features included: (a) the design of an automated salt injection system guaranteeing adequate chloride concentration (350–700 mg/L) in the incoming water to ECR tank that ensured consistent disinfection after electrolysis and allowed to maintain adequate chlorine residuals in the treated water. (b) The implementation of an in situ electrode cleaning mechanism using reverse polarity along with the hopper and suction pipe designs for the bottom of the ECR tank, served to decrease the maintenance requirements with no degradation of electrode performance. This design, informed by the VOF model to determine the optimum level for suction pipe location, prevented sediments flow into the treated water tank with a minimal dead volume of only 0.65% of the total ECR tank. (c) A U‐shaped stirrer to be placed at the center location of the ECR tank was demonstrated by both CFD modeling and laboratory experiment to assure uniform mixing of the ECR contents. We expect that the lessons learned, and design improvement here described will provide valuable input to the development of robust onsite sanitation systems using electrolysis to achieve blackwater treatment safe to discharge for onsite reuse.

## Conflict of interest

The authors have no conflict of interest to declare.

## Supporting information

Supplementary Material

Video S1

## Data Availability

The data that support the findings of this study are available from the corresponding author upon reasonable request.

## References

[wer1374-bib-0001] Cho, K. , & Hoffmann, M. R. (2014). Urea degradation by electrochemically generated reactive chlorine species: Products and reaction pathways. Environmental Science & Technology, 48(19), 11504–11511. 10.1021/es5025405 25219459

[wer1374-bib-0002] Cho, K. , Qu, Y. , Kwon, D. , Zhang, H. , Cid, C. A. , Aryanfar, A. , & Hoffmann, M. R. (2014). Effects of anodic potential and chloride ion on overall reactivity in electrochemical reactors designed for solar‐powered wastewater treatment. Environmental Science & Technology, 48(4), 2377–2384. 10.1021/es404137u 24417418

[wer1374-bib-0003] Cid, C. A. , Jasper, J. T. , & Hoffmann, M. R. (2018). Phosphate recovery from human waste via the formation of hydroxyapatite during electrochemical wastewater treatment. ACS Sustainable Chemistry & Engineering, 6(3), 3135–3142. 10.1021/acssuschemeng.7b03155 29607266 PMC5871340

[wer1374-bib-0004] Cid, C. A. , Qu, Y. , & Hoffmann, M. R. (2018). Design and preliminary implementation of onsite electrochemical wastewater treatment and recycling toilets for the developing world. Environmental Science‐Water Research & Technology, 4(10), 1439–1450. 10.1039/c8ew00209f 33365135 PMC7705125

[wer1374-bib-0005] Hoffmann, M. R. , Aryanfar, A. , Cho, K. , Cid, C. A. , Kwon, D. , & Qu, Y. (2014). Self‐contained, pv‐powered domestic toilet and wastewater treatment system: US Patent App. 14/048,163.

[wer1374-bib-0006] Huang, X. , Qu, Y. , Cid, C. A. , Finke, C. , Hoffmann, M. R. , Lim, K. , & Jiang, S. C. (2016). Electrochemical disinfection of toilet wastewater using wastewater electrolysis cell. Water Research, 92, 164–172. 10.1016/j.watres.2016.01.040 26854604 PMC4773403

[wer1374-bib-0007] Jasper, J. T. , Shafaat, O. S. , & Hoffmann, M. R. (2016). Electrochemical transformation of trace organic contaminants in latrine wastewater. Environmental Science and Technology, 50(18), 10198–10208. 10.1021/acs.est.6b02912 27564843

[wer1374-bib-0008] Jin, H. , Yu, Y. , Zhang, L. , Yan, R. , & Chen, X. (2019). Polarity reversal electrochemical process for water softening. Separation and Purification Technology, 210, 943–949. 10.1016/j.seppur.2018.09.009

[wer1374-bib-0009] Kone, D. (2012). Water, sanitation and hygiene: Reinvent the toilet challenge Paper presented at the meeting abstracts.

[wer1374-bib-0010] Lee, H.‐J. , Hong, M.‐K. , & Moon, S.‐H. (2012). A feasibility study on water softening by electrodeionization with the periodic polarity change. Desalination, 284, 221–227. 10.1016/j.desal.2011.09.001

[wer1374-bib-0011] Min, B. , O'Keeffe, Z. , & Zhang, F. (2017). Whose power gets cut? Using high‐frequency satellite images to measure power supply irregularity. The World Bank.

[wer1374-bib-0012] Palmas, S. , Mascia, M. , Vacca, A. , Mais, L. , Corgiolu, S. , & Petrucci, E. (2018). Chapter 16 ‐ Practical aspects on electrochemical disinfection of urban and domestic wastewater In Martínez‐Huitle C. A., Rodrigo M. A., & Scialdone O. (Eds.), Electrochemical water and wastewater treatment (pp. 421–447). Oxford, UK: Butterworth‐Heinemann.

[wer1374-bib-0013] Park, H. , Choo, K.‐H. , Park, H.‐S. , Choi, J. , & Hoffmann, M. R. (2013). Electrochemical oxidation and microfiltration of municipal wastewater with simultaneous hydrogen production: Influence of organic and particulate matter. Chemical Engineering Journal, 215–216, 802–810. 10.1016/j.cej.2012.11.075

[wer1374-bib-0014] Park, H. , Vecitis, C. D. , & Hoffmann, M. R. (2008). Solar‐powered electrochemical oxidation of organic compounds coupled with the cathodic production of molecular hydrogen. Journal of Physical Chemistry A, 112(33), 7616–7626. 10.1021/jp802807e 18656909

[wer1374-bib-0015] Prüss‐Üstün, A. , Wolf, J. , Corvalán, C. , Bos, R. , & Neira, M. (2016). Preventing disease through healthy environments: A global assessment of the burden of disease from environmental risks. New York, NY: World Health Organization.

[wer1374-bib-0016] Schulze, D. (2008). Powders and bulk solids In Behaviour, characterization, storage and flow (p. 22). Berlin, Germany: Springer.

[wer1374-bib-0017] Spears, D. , Ghosh, A. , & Cumming, O. (2013). Open defecation and childhood stunting in India: An ecological analysis of new data from 112 districts. PLoS One, 8(9), e73784 10.1371/journal.pone.0073784 24066070 PMC3774764

[wer1374-bib-0018] Torotwa, I. , & Ji, C. (2018). A study of the mixing performance of different impeller designs in stirred vessels using computational fluid dynamics. Designs, 2(1), 10.

[wer1374-bib-0019] Varigala, S. K. , Hegarty‐Craver, M. , Krishnaswamy, S. , Madhavan, P. , Basil, M. , Rosario, P. , … Luettgen, M. (2020). Field testing of an onsite sanitation system on apartment building blackwater using biological treatment and electrochemical disinfection. Environmental Science: Water Research & Technology, 6(5), 1400–1411. 10.1039/C9EW01106D

[wer1374-bib-0020] Welling, C. M. , Varigala, S. , Krishnaswamy, S. , Raj, A. , Lynch, B. , Piascik, J. R. , … Grego, S. (2020). Resolving the relative contributions of cistern and pour flushing to toilet water usage: Measurements from urban test sites in India. Science of the Total Environment, 730, 138957 10.1016/j.scitotenv.2020.138957 PMC7272130 32402964

[wer1374-bib-0021] Wendt, H. , & Kreysa, G. (1999). Electrochemical engineering: Science and technology in chemical and other industries. Berlin, Germany: Springer Science & Business Media.

[wer1374-bib-0022] Weres, O. (2009). U.S. Patent 7,494,583.

[wer1374-bib-0023] Weres, O. , & O'Donnell, H. E. (2003). U.S. Patent 6,589,405. U.S. Patent.

[wer1374-bib-0024] WHO, UNICEF JMP (2019). Progress on household drinking water sanitation and hygiene 2000–2017: Special focus on inequalities. New York, NY: United Nations Children's Fund and World Health Organization.

