# Supplementary material for: Optimal design of an electrochemical reactor for blackwater treatment
Source: Water Environ Res. 2020 Jul 5;93(1):148–58. doi: 10.1002/wer.1374 (PMC7818490; doi:10.1002/wer.1374)
Supplement: Supplementary file 1 — Supplementary Material [file WER-93-148-s001.docx]

Title: Optimal Design of an Electrochemical Reactor for Blackwater Treatment

Authors: Siva Varigala^1,2^, Srinivas Krishnaswamy^1^, Chandra P. Lohia^2^, Meghan Hegarty-Craver^3^, Sonia Grego^3,4^, Michael Luettgen^5^, Clement A. Cid*^6^

^1^ Dept. Chem. Engineering, BITS Pilani K K Birla Goa campus, Goa 403726 (India)

^2^ ITC-Kohler Co., Pune, Maharashtra 411013 (India)

^3^ RTI International, Research Triangle Park, NC 27709 (USA)

^4^ Center for WaSH-AID, Duke University, Durham, NC 27708 (USA)

^5^ Kohler Co., Kohler, WI 53044 (USA)

^6^ California Institute of Technology, Pasadena CA 91125 (USA)

*Corresponding author: ccid@caltech.edu

# SUPPLEMENTARY INFORMATION

## Figures


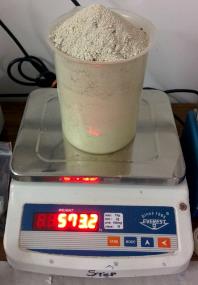


*Figure S1: 573 g of dried precipitate were collected after less than one week of operation of the CLASS v1 system in Unit A.*


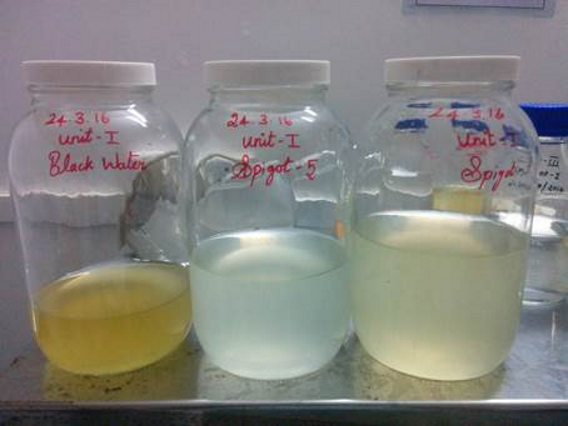


*Figure S2: Comparison of the water at various points in the treatment process. Spigot 3 is water just prior to electrolysis treatment. Spigot 5 is water after ECR treatment and filtration. Spigot 1 is treated water collected in the overhead treated water tank*

*Figure S3: Standard design charts to (A) determine the optimum angle of the planar hopper with the vertical and (B) aids to determine the outlet size (Schulze, 2008).*


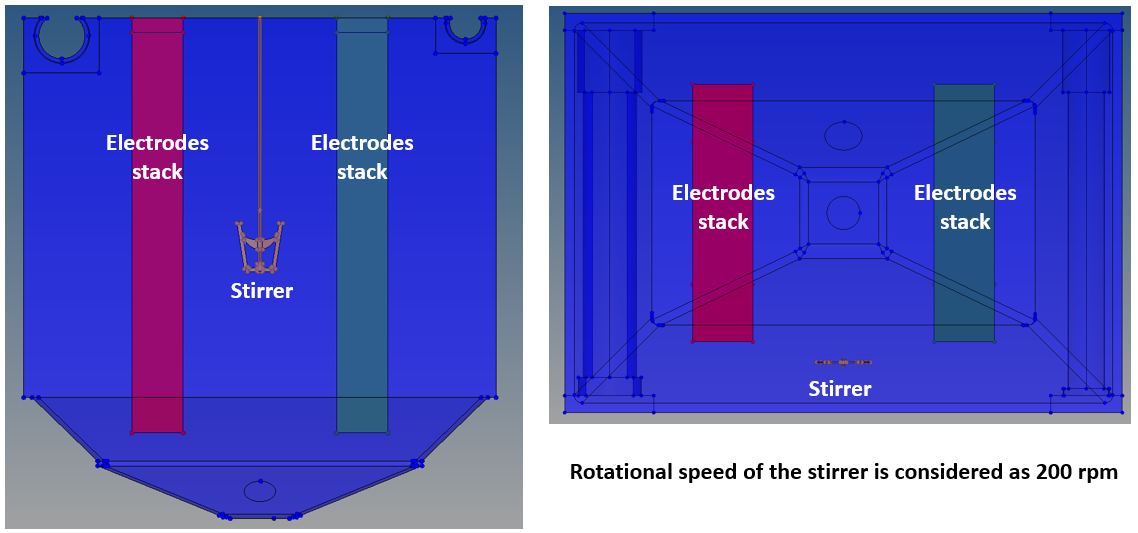


Figure S4: Representation of the U-shaped stirrer model


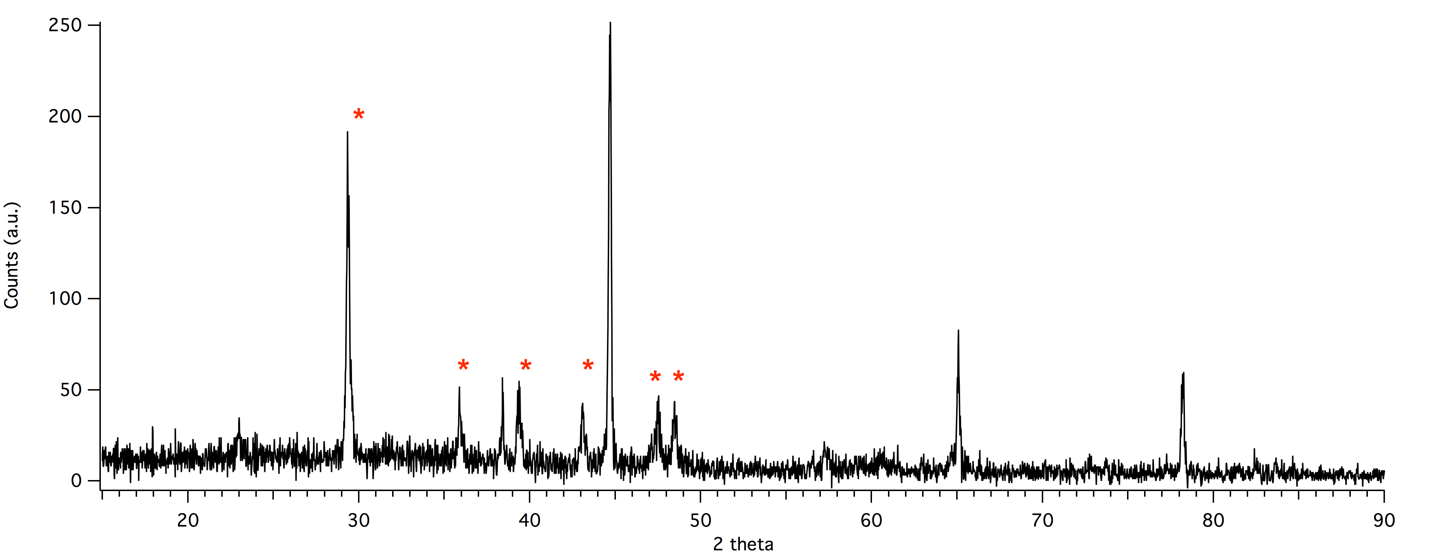


Figure S5: X-ray diffraction spectrum of collected precipitate. Peaks associated with calcium carbonates are annotated with an asterisk.


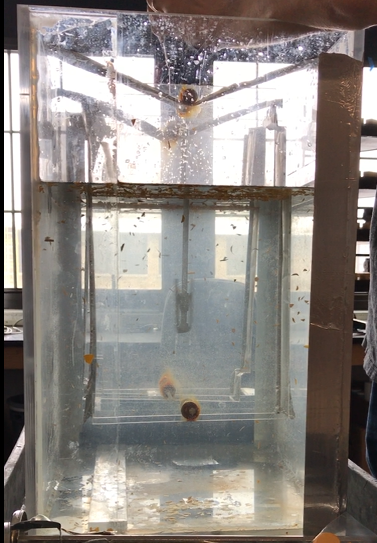

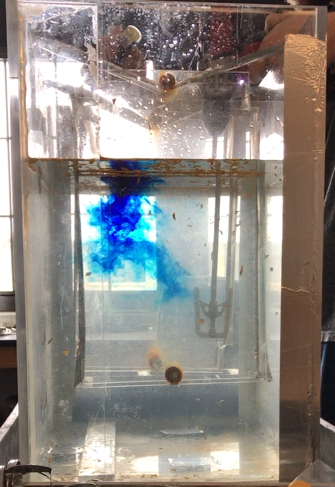


Figure S6: Screenshots of videos of Lab tests confirming the CFD results for the U-shaped stirrer. Left: U-shaped stirrer located in the center showing the mixing distribution of the pieces of paper Right: dispersion of methylene blue dye in water due to stirrer rotation showing the mixing gradients in the reactor volume. Link to video available in Supplementary Material.

## Tables

Table S1: Water quality analysis of CLASS v1 from site A and site B.

| **Parameter** | **Unit A** | | | **Unit B** | | |
| --- | --- | --- | --- | --- | --- | --- |
|  | **Blackwater** | **Treated** | **Removal** | **Blackwater** | **Treated** | **Removal** |
| **TSS (mg/L)** | **78** ± 20  (n=20) | **48** ± 12  (n=18) | **38%** | **58** ± 24  (n=24) | **9** ± 10  (n=25) | **84%** |
| **Cl^-^**  **(mg/L)** | **476** ± 123 (n=20) | **555** ± 101 (n=18) | **--** | **598** ± 201 (n=28) | **504** ± 214 (n=28) | **--** |
|  | **161** ± 47 (n=7)***** | **--** | **--** | **196** ± 50 (n=6)***** | **--** | **--** |
| **Alkalinity (mg CaCO_3_/L)** | **905** ± 101  (n=19) | **360** ± 158  (n=18) | **60%** | **279** ± 66  (n=28) | **11** ± 24  (n=27) | **96%** |
| **COD (mg/L)** | **579** ± 131  (n=18) | **433** ± 188  (n=18) | **25%** | **383** ± 114  (n=24) | **261** ± 101  (n=27) | **32%** |
| **TKN (mg/L)** | **161** ± 18  (n=18) | **110** ± 56  (n=18) | **32%** | **87** ± 28  (n=28) | **13** ± 14  (n=27) | **85%** |
| **NH_3_  (mg/L)** | **153** ± 12  (n=18) | **89** ± 38  (n=18) | **42%** | **68** ± 13  (n=27) | **7** ± 10  (n=27) | **90%** |

Note: “n” stands for the number of samples
